# Correction: Breaking the spiral of silence: News and social media dynamics on sexual abuse scandal in the Japanese entertainment industry

**DOI:** 10.1371/journal.pone.0317150

**Published:** 2025-01-02

**Authors:** Tsukasa Tanihara, Mitsuki Irihara, Taichi Murayama, Mitsuo Yoshida, Fujio Toriumi, Kunihiro Miyazaki

The Funding statement for this paper is incorrect. The correct Funding statement is as follows: This work is supported in part by the Research Institute of Science and Technology for Society, Japan Science and Technology Agency (JST RISTEX) JPMJRS23L1 awarded to F.T. The funders have no role in study design, data collection and analysis, decision to publish, or preparation of the manuscript.

## References

[1] Tanihara T, Irihara M, Murayama T, Yoshida M, Toriumi F, Miyazaki K (2024) Breaking the spiral of silence: News and social media dynamics on sexual abuse scandal in the Japanese entertainment industry. PLOS ONE 19(6): e0306104. doi: 10.1371/journal.pone.0306104 38935809 PMC11210866

