# Correction: Breaking the spiral of silence: News and social media dynamics on sexual abuse scandal in the Japanese entertainment industry

**DOI:** 10.1371/journal.pone.0319695

**Published:** 2025-02-26

**Authors:** The PLOS One Staff

There is an error in affiliation 5 for author Fujio Toriumi. The correct affiliation 5 is: University of Tokyo, Tokyo, Japan.

The publisher apologizes for the error.
