# Supplementary material for: Breaking the spiral of silence: News and social media dynamics on sexual abuse scandal in the Japanese entertainment industry
Source: PLoS One. 2024 Jun 27;19(6):e0306104. doi: 10.1371/journal.pone.0306104 (PMC11210866; doi:10.1371/journal.pone.0306104)
Supplement: S1 Table — (PDF) [file pone.0306104.s001.pdf]

Rank	Words (ja)	Words (en)	Count	Sexual abuse?	Accuracy
1	加害	violence	68,563	yes	100%
2	性	sex	41,347		
3	問題	issue	39,020		
4	ジャニーズ事務所	johnny's office	24,505		
5	ジャニー喜多川	johnny kitagawa	12,521		
6	社長	president	10,961		
7	報道	press	10,883		
8	謝罪	apology	9,947		
9	元	former	7,875		
10	被害者	victim	6,356	yes	100%
11	声	voice	4,874		
12	事務所	office	4,827		
13	被害	damage	4,590	yes	100%
14	櫻井翔	sho sakurai	4,453		
15	文春	bunshun	4,387		
16	賛同	endorsement	4,339		
17	告発	accusation	4,180	yes	100%
18	お願い	request	4,131		
19	言及	mention	3,870		
20	疑惑	allegation	3,831	yes	84%
21	ジャニーズJr.	johnny's jr.	3,744		
22	番組	tv program	3,694		
23	検証	verification	3,407	yes	97%
24	告白	confession	3,175	yes	81%
25	日本	japan	2,938		
26	無視	ignorance	2,870		
27	今	now	2,828		
28	人	people	2,821		
29	性暴力	sexual violence	2,786	yes	100%
30	テレビ局	tv station	2,724		

Table S1. The top 50 frequent nouns that appear in X posts related to Johnny's that include "性加害" (sexual abuse). English translations by the authors. Count indicates the number of occurrences in the data. The column Sexual abuse indicates the authors' judgment in picking up candidates for words related to "sexual abuse" (we regarded "性" and "加害" as one word). Finally, we checked 100 posts containing each candidate word and adopted words with 100% accuracy (Accuracy column) related to the case of Johnny Kitagawa.
