# Supplementary material for: Breaking the spiral of silence: News and social media dynamics on sexual abuse scandal in the Japanese entertainment industry
Source: PLoS One. 2024 Jun 27;19(6):e0306104. doi: 10.1371/journal.pone.0306104 (PMC11210866; doi:10.1371/journal.pone.0306104)
Supplement: S2 Table — (PDF) [file pone.0306104.s002.pdf]

Cluster	Ratio
Politics1	32.2%
Fan1 (Pro-Johnny)	14.8%
Politics2	14.5%
Fan2 (Anti-Johnny)	11.6%
Fan3 (Anti-Johnny)	11.4%
Sum	84.5%

Table S2. Cluster names obtained from network clustering and the percentage of size each cluster occupies in the network.
