# Supplementary material for: Breaking the spiral of silence: News and social media dynamics on sexual abuse scandal in the Japanese entertainment industry
Source: PLoS One. 2024 Jun 27;19(6):e0306104. doi: 10.1371/journal.pone.0306104 (PMC11210866; doi:10.1371/journal.pone.0306104)
Supplement: S3 Table — Representativeness were calculated by Tf-Idf. (PDF) [file pone.0306104.s003.pdf]

Topics	Representative words (en)
Press conference	surmise, press conference, TV program, NHK, president, comment, TV, appointment, press conference, TV company
News and fact-finding	accusation, fact, trial, evidence, testimony, Bunshun, person concerned, fact-finding, police, fan
Responsibility	ceo, remedy, company, Julie, corporation, compensation, press conference, response, responsibility, apology
Johnny and crime	boy, entertainment industry, male, female, sexual abuse, US military, self, pedophilia, child, sexual
Mass media's surmise	mass media, incident, accusation, surmise, crime, TV company, entertainment industry, TV, responsibility, concealment
Fans' feeling	fan, company, accusation, feelings, company, appointment, criticism, job, support, remark

Table S3. Result of topic modeling, indicating topic names of post contents and their representative words (English). The texts are translated by DeepL translator (`https://www.deepl.com/translator`).
