# Supplementary material for: Breaking the spiral of silence: News and social media dynamics on sexual abuse scandal in the Japanese entertainment industry
Source: PLoS One. 2024 Jun 27;19(6):e0306104. doi: 10.1371/journal.pone.0306104 (PMC11210866; doi:10.1371/journal.pone.0306104)
Supplement: S4 Table — (PDF) [file pone.0306104.s004.pdf]

Topics	Representative words (ja)
Press conference	忖度, 会見, 番組, nhk, 社長, コメント, テレビ, 起用, 記者会見, テレビ局
News and fact-finding	告発, 事実, 裁判, 証拠, 証言, 文春, 当事者, 認定, 警察, ファン
Responsibility	社長, 救済, 会社, ジュリー, 企業, 補償, 会見, 対応, 責任, 謝罪
Johnny and crime	少年, 芸能界, 男性, 女性, 性的虐待, 米軍, 自ら, 小児性愛, 子供, 性的
Mass media's surmise	マスコミ, 事件, 告発, 忖度, 犯罪, テレビ局, 芸能界, テレビ, 責任, 隠蔽
Fans' feeling	ファン, 企業, 告発, 気持ち, 会社, 起用, 批判, 仕事, 応援, 発言

Table S4. Result of topic modeling, indicating topic names of post contents and their representative words (original).
