# Supplementary material for: Breaking the spiral of silence: News and social media dynamics on sexual abuse scandal in the Japanese entertainment industry
Source: PLoS One. 2024 Jun 27;19(6):e0306104. doi: 10.1371/journal.pone.0306104 (PMC11210866; doi:10.1371/journal.pone.0306104)
Supplement: S5 Table — (PDF) [file pone.0306104.s005.pdf]

Cluster	Representative words (original)
Politics1	政治, 反対, 日本, 映画, 音楽, 応援, 社会, 趣味, 原発, 自由
Fan1 (Pro-Johnny)	応援, ファン, 箱推し, 日本, johnny, 松本潤, kinki, 相葉, 木村拓哉, news
Politics2	日本, 日本人, 政治, 趣味, 反対, 応援, アニメ, 賛同, ゲーム, 反日
Fan2 (Anti-Johnny)	smap, 応援, アニメ, ファン, 映画, 趣味, 音楽, king, ゲーム, 多め
Fan3 (Anti-Johnny)	smap, king, 応援, ara, prince, 紫耀, prism, 箱推し, ファン, 新しい地図

Table S5. Cluster names of network clustering and their representative words (original) from profiles of users in each group. Representativeness were calculated by Tf-Idf.
