# Supplementary material for: Breaking the spiral of silence: News and social media dynamics on sexual abuse scandal in the Japanese entertainment industry
Source: PLoS One. 2024 Jun 27;19(6):e0306104. doi: 10.1371/journal.pone.0306104 (PMC11210866; doi:10.1371/journal.pone.0306104)
Supplement: S6 Table — (PDF) [file pone.0306104.s006.pdf]

Cluster	Rank	Texts
Politics1	1	Press conference ends. Conclusion: We will not change the name of Johnny's Office, and we will not give up our shares. We will not set up a third-party committee. We will compensate the victims, but nothing concrete has been decided yet - in other words, "Johnny's Office will not change. <URL>
	2	Interviews with people involved in the issue of sexual assault by former Johnny's Office president Janie Kitagawa have revealed that a working group of the United Nations Human Rights Council will visit Japan later this month to conduct interviews with parties who claim to have been victimized. Experts from the Human Rights Council, which addresses human rights violations in various countries and urges improvements, have begun to grasp the actual situation. <URL>
	3	The special report on the issue of Janie Kitagawa's sexual assault in the news special. It was very heavy. In a video 34 years ago, former Four Leaves member Kouji Kita angrily appealed to both Mr. Janney and Ms. Mary, who left his surname perpetration silent, to "stop deceiving children and their parents" and to stop. <URL><URL>
	4	The importance of speaking out. The denunciation that "(Mr. Janey) can't tell us after he's dead" is misguided. Mr. Hattori and Ms. Matsuzaki made a courageous choice to "speak out while (he) was alive. Actor Kichiji Hattori testified that he was sexually harmed by Mr. Janney in his room 70 years ago：The Asahi Shimbun Digital <URL>
	5	It is not only Johnny's that TV is not reporting. They didn't report the Kihara case, the invoice, the abolition of the insurance card, the former Unification Church, etc. They didn't report the ugly news about the Korean Minister of Justice in 2019 when Typhoon No. 19 caused extensive damage in Boso, they didn't report the 2012 rally against nuclear reactors in front of the National Diet, and they didn't report the "Japanesesque" rally against the nuclear reactors in Japan in 2011 when the Japanese government was trying to stop the nuclear reactors in Japan. <URL>
Fan1	1	Dr. Yosuke Naito: "Regarding the Johnny's issue, we have to be careful. Comfort women and activists have entered the Penlight, a group that calls itself a fan group, and they are asking the government to give 3% of the sales of the Johnny's office to relief for victims, etc. This is a dangerous move. This is a dangerous move." Naito-san is flying in this morning #GoodMorningTerachan
	2	The sports newspaper is reporting 10 billion yen in total damages for the sexual assault issue at the Johnny's office!, but, um, damages for torts disappear after 3 years...
	3	Okay? Please calm down and listen to me. The report issued by the third-party committee on the Johnny's issue did not confirm the enrollment of the members on the victim's side, and it also clearly stated that it did not investigate their testimonies of sexual harm "because of the sensitive nature of the content <URL><URL>
	4	Summary of the current status of the Johnny's issue (23/8/29) *All are exposed by Johnny's fans *Till now, there is no evidence of sexual harm and even the police and judiciary have not reported it <URL>
	5	Junya Hiramoto "If we get 3% from Johnny's every year, we can save quite a few victims lol" He is planning to rip off Johnny's money forever #Janney's office sexual assault problem #WeSupportJohnny'sOffice <URL>
Politics2	1	The media, which is a bystander and an accomplice of sexual assault, is really too much of a devil.
	2	Please check out the gratifying tweets by Ms. Minori Kitahara, an endorser of "PENLIGHT: A Group to Reveal the Sexual Abuse of the Johnny's Office" founded by Johnny's fans, director of the Pornography and Prostitution Problem Study Group, adult goods store owner, and famous for taking nude photos at Yasukuni Shrine. <URL>
	3	What an extraordinary demand. What are you talking about? Why would the government get involved and give out the people's tax money? This is the problem with the japanese and those involved. The japanese should be the only ones to pay for the crime and compensate for it. Did the government order you to join Johnny's? Former Johnny's Jr. appealed to the Diet: "The government should get involved and help the victims." <URL>
	4	After all, the former Johnny's who are suing for sexual harm after Janney's death must have accepted to be Janney's toys in order to be stars. Of course Janney is sickening, but the former Janneys themselves who are victims are sickening too. There is no obligation or reason for the taxpayers to help them, so I hope the parties involved will resolve this issue.
	5	NHK apologized for the Johnny's sexual assault case, saying, "It has often been reported in weekly magazines, etc., and a court decision has been finalized, but NHK was not aware of this issue at the time and has not covered it since then. In short, NHK did not report the inconvenient news even though it knew about it. Hi, it's the same now. Please return the subscription fee. <URL>
Fan2	1	[Breaking News] Tokio Marine & Nichido Fire Insurance Considering Termination of Contract with Masaki Aiba, a Major Non-Life Insurance Company <URL>In response to the sexual assault issue by the late Johnny Kitagawa, founder of the Johnny's Office, Tokio Marine & Nichido Fire Insurance, a major non-life insurance company, is considering terminating its contract with Masaki Aiba, who belongs to the same office. The company is considering terminating the contract with Masaki Aiba, who belongs to the same agency.
	2	Asahi G will not use Johnny's in advertisements in the future. https://t.co/3yyRRtVdoB In response to the Johnny's office sex assault issue, the company announced that it "will not develop any new advertisements or new sales promotions featuring talent from the Johnny's office in the future. The current contract will be terminated upon expiration.
	3	In response to the issue of a series of voices from male former talents of the Johnny's Office and others claiming sexual assault by former president Janie Kitagawa, who died four years ago, the Johnny's Office released a video featuring the current president on March 14. <URL>#nhk_video <URL>
	4	[Approx. 16,000 signatures] Johnny's fans mailed signatures, demanding verification from the office <URL>This is in response to an accusation by a former Johnny's Jr. man who claims he was sexually victimized by former president Janie Kitagawa. The women who are fans held a press conference and revealed that they mailed the signatures they had collected demanding verification of the assaults.
	5	The news23 program featured the allegations of sexual assault by Johnny's Jr. and made an unusual self-criticism. <URL>For about 10 minutes, the program reported the testimonies of the victims and asked, "How much have the media organizations reported on this kind of damage? There is a current situation that has not been reported, at least in our program," he said, touching on the responsibility of the media. <URL>
Fan3	1	Good evening. The first half of this week's Special Report on June 17 (Sat.) will focus on "Verification: The Origin of Janney's Sexual Assault". Why was the spread of the damage not stopped? What is the responsibility of broadcasters? Please watch. #TBS #JNN <URL>
	2	The July 4 issue of #NikkanGendai. The BBC's documentary on the late Janie Kitagawa's sexual assault has triggered shockwaves, including accusations of real names. This time, Kichiji Hattori, the second son of National Honor Award-winning composer Ryoichi Hattori, confesses the sexual assault he suffered from Mr. Janney. For more information, please see the special feature on page 14! <URL>
	3	Interviews with people involved in the issue of sexual assault by former Johnny's Office president Janney Kitagawa have revealed that a working group of the United Nations Human Rights Council will visit Japan later this month to conduct interviews with parties who have complained of victimization. Experts from the Human Rights Council, which addresses human rights violations in various countries and urges improvements, have begun to grasp the actual situation. <URL>
	4	The press conference by the Johnny's office is "brilliantly theatrical" and "bankrupt" = Mr. Yoshitsugu Hattori, Association of Victim Parties <URL>"New President Higashiyama, Julie and Inohara are all brilliantly theatrical" "Suguru Shirahase (vice president) who cannot act is not in the press conference" "Why did you hide him, come out. Come out" #johnnys #johnnys <URL>
	5	I watched the press conference from the beginning to the end, and I was impressed by the "childishness" of the Johnny's office: no one takes "responsibility", they don't even change the name of the offender, they don't know, they don't care, they talk as if they were the victims, they talk about "sexual damage", but they make up for it through "entertainment"........ The press conference gave the impression of the "childishness" of the Johnny's office.

Table S6. Clusters and the posts each group shared most (English). The texts are translated by DeepL translator (`https://www.deepl.com/translator`).
