# Supplementary material for: Breaking the spiral of silence: News and social media dynamics on sexual abuse scandal in the Japanese entertainment industry
Source: PLoS One. 2024 Jun 27;19(6):e0306104. doi: 10.1371/journal.pone.0306104 (PMC11210866; doi:10.1371/journal.pone.0306104)
Supplement: S7 Table — (PDF) [file pone.0306104.s007.pdf]

Cluster	Rank	Texts
Politics1	1	記者会見終了。 結論：ジャニーズ事務所の名前は変えず、株も手放しません。第三者委員会も設置しません。被害者補償はするけど具体的なことはまだ何も決めてないです——すなわち「ジャニーズ事務所は変わりません」ということ。... <URL>
	2	ジャニーズ事務所のジャニー喜多川前社長による性加害問題で、国連人権理事会の作業部会が今月下旬に来日し、被害を訴える当事者への聞き取り調査に乗り出すことが、関係者らへの取材で分かった。各国の人権侵害を取り上げ、改善を促す人権理の専門家が実態把握に動き出した。<URL>
	3	報道特集のジャニー喜多川氏の性加害問題の特集記事。とても重かった。元フォーリーブスの北公次さんは、34年前のビデオで、ジャニー氏にも彼の姓加害を黙殺放置したメリー氏にも「いい加減、子どもやその親を騙すのをやめてくれよ」と怒りを込め、やめるよう訴えていた... <URL><URL>
	4	声を上げることの大切さ。 「（ジャニー氏が）死んでから言うなんて」という糾弾はお門違い。服部さんと松崎さんは「（ご自分が）生きているうちに言う」という勇気ある選択をしたのだ。 「70年前、ジャニー氏から自室で性被害」俳優の服部吉次さんが証言：朝日新聞デジタル <URL>
	5	TVが報道してないのはジャニーズだけではない。木原事件も、インボイスも、保険証廃止のマイナカードも、旧統一教会についても、あんまり報道してない。２０１９年、台風１９号で房総で甚大な被害が出ていた時も韓国の法務大臣の醜聞を報道。２０１２年の国会前の原発再稼働反対集会も報道しなかった。
Fan1	1	内藤陽介先生「ジャニーズ問題、気をつけなければならないのは、ペンライトというファンを自称する団体に慰安婦問題や活動家が入り込んで国に対してジャニーズ事務所の売り上げの3%を被害者救済にあてろ等と言っている。これは危険な動き」 内藤さん朝から飛ばしてくれてます #おはよう寺ちゃん
	2	ジャニーズ事務所の性加害問題、スポーツ新聞で損害賠償総額100億円!って報じてるけど、あのー、不法行為の損害賠償って3年で消えるんですけど
	3	いいですか？落ち着いて聞いてください ジャニーズ問題の第三者委員会が出した報告書には、被害者側のメンバーの在籍確認もしておらず、また彼らの性被害の証言を調査することは「センシティブな内容なので調査はしなかった」と明言されていました。 <URL><URL>
	4	ジャニーズ問題の現状まとめ（23/8/29） ※全部ジャニーズファンによって暴かれる ※現在まで性被害の証拠は無く、警察や司法すら届け出していません... <URL>
	5	平本淳也 「毎年ジャニーズから3%もらえれば相当数の被害者が救えるんじゃないか笑」 未来永劫ジャニーズの金をむしり取るつもりでいらっしゃる #ジャニーズ事務所性加害問題 #ジャニーズ事務所を応援します
Politics2	1	車傷付けて金儲けしてたビッグモーターをあれだけぶっ叩いておきながら、少年を傷付けて金儲けしてたジャニーズを大して叩かず、挙げ句の果てには国民の血税で助けろとか喚いてる性加害の傍観者、共犯者の鬼畜マスコミ、マジでやばすぎだろ。
	2	ジャニーズファンで立ち上げた「PENLIGHT ジャニーズ事務所の性加害を明らかにする会」の賛同人、ポルノ・買春問題研究会理事、アダルトグッズショップ経営者、靖国神社で全裸写真撮影で有名な北原みのりさんによるありがたいツイートをご確認下さい。 <URL>
	3	何と異常な要求。何を言ってんの？なぜ国が関与して国民の税金を出すの？これはジャニーズとその関係者の問題だろ。罪を償うのも賠償もジャニーズだけでやるべきだろ。 国からジャニーズに入れと命令でもされたのか？ 元ジャニーズJr.が国会で訴え「国が関与し被害者救済を」 <URL>
	4	結局、ジャニーの死後に性被害を訴えている元ジャニーズも、スターになりたいが為にジャニーのおもちゃになる事を受け入れていたんだろうね。ジャニーはもちろん気持ち悪いが、被害者面してる元ジャニーズ自体も気持ち悪い。税金で助ける義務も道理もないので、当事者で解決してもらいたい。
	5	ジャニーズ性加害事件にNHKは「これまでも週刊誌等でたびたび報じられ､裁判でも判決が確定していましたがNHKは当時この問題に認識が薄く､その後も取り上げる事はありませんでした」とお詫び。要するに都合の悪いニュースは知ってても報じませんでした､と。ハイ､今も同じですね。受信料お返し下さい <URL>
Fan2	1	【速報】相葉雅紀との契約解除を検討、損害保険大手の東京海上日動火災保険 <URL>ジャニーズ事務所創業者の故ジャニー喜多川氏による性加害問題を受け、損害保険大手の東京海上日動火災保険が、同事務所に所属する相葉雅紀との契約解除を検討していることが分かった。
	2	【発表】アサヒG、今後ジャニーズを広告に起用せず <URL>ジャニーズ事務所の性加害問題を受け「今後、ジャニーズ事務所のタレントを起用した広告や新たな販促は展開しない」と明らかにした。現時点の契約は満了をもって解除するという。
	3	ジャニーズ事務所の元所属タレントの男性などから、4年前に亡くなったジャニー喜多川前社長による性被害を訴える声が相次いでいる問題で、ジャニーズ事務所は14日、今の社長が出演する動画を公開しました。 <URL>#nhk_video <URL>
	4	【約1万6千筆】ジャニーズファンが署名郵送、事務所に検証求める <URL>ジャニー喜多川前社長から性被害を受けたとする元ジャニーズJr.の男性の告発を受けたもの。ファン有志の女性らは記者会見を開き、加害の検証などを求めて集めた署名を郵送したと明らかにした。
	5	【11日放送】news23がジャニーズ性加害疑惑を特集、異例の自己批判 <URL>約10分間にわたり被害者の証言などを伝え、「報道機関がどれだけ、こうした被害を報道してきたのか。少なくとも私たちの番組ではお伝えしてこなかった現状があります」とメディアの責任に触れた。 <URL>
Fan3	1	こんばんは。 今週6月17日（土）の #報道特集 前半の特集は 『検証・ジャニー氏性加害の原点』 ジャニー氏による性加害は一部のメディアで60年以上前から 報じられてきた。 なぜ被害の拡大は食い止められなかったのか？ 放送事業者の責任とは？ 是非ご覧ください。 #TBS #JNN <URL>
	2	7/4(火)発行の #日刊ゲンダイ です。 英ＢＢＣが報じた故ジャニー喜多川氏の性加害問題のドキュメンタリーを契機に、実名告発など衝撃が広がっている。今回、国民栄誉賞受賞作曲家・服部良一氏の次男、服部吉次さんが、ジャニー氏から受けた性加害を告白する。 詳しくは、14面特集をご覧ください!<URL>
	3	ジャニーズ事務所のジャニー喜多川前社長による性加害問題で、国連人権理事会の作業部会が今月下旬に来日し、被害を訴える当事者への聞き取り調査に乗り出すことが、関係者らへの取材で分かった。各国の人権侵害を取り上げ、改善を促す人権理の専門家が実態把握に動き出した。 <URL>
	4	ジャニーズ事務所の会見は「見事にお芝居」「破綻している」＝被害者当事者の会・服部吉次氏 <URL>「東山新社長もジュリーも井ノ原も、見事な芝居」 「芝居ができない白波瀬傑（副社長）が会見に出てきていない」 「なぜ彼を隠したのか、出てこい」 #ジャニーズ #johnnys <URL>
	5	会見を最初から最後まで見たけど、誰も「責任」を取らない、犯罪者の名前の社名も変えない、知らぬ、存ぜぬ、「寝耳に水」だった自分たちもまるで被害者のような物言い、「性被害」の話なのに「エンタメで挽回」とワケのわからない決意表明...とジャニーズ事務所の「幼稚さ」を印象付けた会見だった。

Table S7. Clusters and the posts each group shared most (original).
