# Supplementary material for: Breaking the spiral of silence: News and social media dynamics on sexual abuse scandal in the Japanese entertainment industry
Source: PLoS One. 2024 Jun 27;19(6):e0306104. doi: 10.1371/journal.pone.0306104 (PMC11210866; doi:10.1371/journal.pone.0306104)
Supplement: Attachment — Submitted filename: Response_to_Reviewers_km.docx [file pone.0306104.s008.docx]

Response to Editors and Reviewers

**Response to Editors**

1. Please ensure that your manuscript meets PLOS ONE's style requirements, including those for file naming. The PLOS ONE style templates can be found at

https://journals.plos.org/plosone/s/file?id=wjVg/PLOSOne_formatting_sample_main_body.pdf and

<https://journals.plos.org/plosone/s/file?id=ba62/PLOSOne_formatting_sample_title_authors_affiliations.pdf>

**Response:** We rechecked the style requirement. We fixed all headings to be sentence case; unified Figure references to Fig X format.

2. In your Methods section, please include additional information about your dataset and ensure that you have included a statement specifying whether the collection and analysis method complied with the terms and conditions for the source of the data.

**Response:** Based on this point, we have added the following text to the Data Collection chapter:
It should be noted that the collection and analysis of these data comply with the terms of use of the respective data providers. Especially, social media data is not displayed in such a way as to reveal personal information.

3. Please include a separate caption for each figure in your manuscript.

**Response:** We included captions of each figure in the manuscript.

4. Please include captions for your Supporting Information files at the end of your manuscript, and update any in-text citations to match accordingly. Please see our Supporting Information guidelines for more information: <http://journals.plos.org/plosone/s/supporting-information>.

**Response:** We added the captions for your Supporting Information at the end of your manuscript. Also, we modified the numbering of tables (added “S” to each table number) in Supplementary materials.

5. Please review your reference list to ensure that it is complete and correct. If you have cited papers that have been retracted, please include the rationale for doing so in the manuscript text, or remove these references and replace them with relevant current references. Any changes to the reference list should be mentioned in the rebuttal letter that accompanies your revised manuscript. If you need to cite a retracted article, indicate the article’s retracted status in the References list and also include a citation and full reference for the retraction notice.

**Response:** We reviewed the reference list complete and correct. We did not find the papers that have been retracted in the list.

**Response to Reviewers**

1. The research exhibits innovation and is well articulated. However, I suggest that the authors explicitly highlight the novelty and social implications of the study within the manuscript.

**Response:** The novelty of this paper is as follows: This paper quantitatively elucidates the relationship between social media and the Spiral of Silence Theory, a topic that has recently been brought into discussion, by comprehensively and chronologically analyzing the discourse across multiple mass media (TV, newspapers, tabloids, magazines, online news) and social media using a large-scale dataset. The results reveal that social media can break the silence even if the mainstream media remains silent, given a trigger. Furthermore, it was found that reversing the majority and minority leads to the emergence of an opposition to the new majority, and this opposition can be amplified.

This has been added to the latter part of the Literature Review and the Discussion sections. Specifically, for the latter, it has been added as the third item in the section on Social Media and the Spiral of Silence within the Discussion section.

Regarding social implications, a chapter titled "Implications for the Media Industry" has been established to discuss this matter. By enriching the description in this part, the social implications have been further emphasized.

2. While the literature review is comprehensive, I recommend incorporating a section addressing the impact of social media on breaking the spiral of silence. For example, consider including a chapter discussing how social media platforms undermine the spiral of silence by facilitating the expression of diverse voices and opinions: Laor, T. (2023), titled "Breaking the Silence: The Role of Social Media in Fostering Community and Challenging the Spiral of Silence," published in the Online Information Review.

**Response:** In light of your comments, a section titled "The Impact of Social Media on the Spiral of Silence Theory" has been added to the Literature Review, where we discuss based on the literature. In this study, we consider that the mechanism by which social media breaks the Spiral of Silence lies in the reduction of the fear of isolation, brought about by being surrounded by like-minded opinions in the online space. The newly added section discusses this theoretically based on the literature and utilizes it in setting the research question. Additionally, it emphasizes that, unlike previous studies, which were limited by partial data use, interviews, and simulations, this paper is a comprehensive and chronological study using a large-scale dataset.

3. The research results are noteworthy and intriguing. However, the manuscript lacks explicitly stated research hypotheses and questions. It is essential to formulate these elements based on the insights gained from the literature review.

**Response:** In the final part of the Literature Review, the following research question was articulated:

RQ: In the context of sexual abuse issues that were silenced by mass media, what role has social media played in shaping public opinion?

On the other hand, this paper is not of a hypothesis-testing nature but rather explores and describes big data in an exploratory and comprehensive manner. Therefore, no hypothesis was established.

4. Integrating research questions and hypotheses derived from the literature review will not only enhance the overall quality of the paper but also contribute to a more enriched discussion.

**Response:** By establishing the following research question, the discussion has been enriched:

RQ: In the context of sexual abuse issues that were silenced by mass media, what role has social media played in shaping public opinion?

The role of social media was not only to break the silence but also to create a complex situation where it reversed the majority and minority, leading to the emergence of an opposition to the new majority and amplifying it. To elucidate this, a third item has been added to the section on Social Media and the Spiral of Silence in the Discussion section.

In addition, we should note that in the annotation indicating statistical tests in Fig. 2, we did not erase the one indicating “no significance” in the figure, so we have erased it. However, this does not affect the overall argument.
